# Constant mixing temperature test of a fin-and-tube latent heat thermal energy storage

**DOI:** 10.1038/s41598-022-24990-0

**Published:** 2022-12-05

**Authors:** Petr Jančík, Michal Schmirler, Tomáš Hyhlík, Jakub Suchý, Pavel Sláma, Petr Prokop, Viktor Syrovátka

**Affiliations:** Faculty of Mechanical Engineering, Department of Fluid Dynamics and Thermodynamics, CTU in Prague, Technická 4, 160 00 Prague, Czech Republic

**Keywords:** Energy science and technology, Engineering

## Abstract

Thermal energy accumulation is one of the ways how to optimize heat production processes and how to balance the supply and demand of heat in distribution systems. This article presents a design of a fin-and-tube latent heat thermal energy storage (LHTES), which combines high thermal energy storage density and scalability. A computational model that used lumped heat capacities was tuned using the experimental data. The numerical model proved to be simple yet precise. A new constant mixing temperature test was designed and performed with the LHTES. Unlike standard constant flow rate charge/discharge test, this test provided valuable information about what to expect in the real-life operation conditions. From the tests and data from simulations, it was concluded that the LHTES would perform better in terms of its capacity utilization if it operated at lower output power than in the laboratory circuit. This indicates that a smaller, and thus more cost-effective, LHTES could be employed in the laboratory circuit with virtually the same utility to the system if its heat transfer characteristics were improved.

## Introduction

The demand for heat in housing, industry, agriculture, and other facilities naturally varies. There are seasonal differences and daily patterns, which lead to huge differences between consumption peaks and valleys^1,2^. Unfortunately, the supply and demand for heat usually do not meet at a given time. At times of heat abundance from solar systems, the demand for heat is very low, and vice versa^3^. Of course, heat sources based on fuel combustion operate independently of weather conditions, but they have other drawbacks. Heat and electricity are often co-generated to increase overall fuel efficiency. Cogeneration is, without a doubt, a beneficial concept, but it may lead to a mismatch between heat production and consumption again. Moreover, fuels are costly nowadays and generate (at least) local emissions. To run these heat sources economically and ecologically, their heating power output should be close to the nominal value. However, this is often not the case because the heat sources are designed to cover the maximal demand, so they run on relatively low power settings most of the time.

Of course, heat accumulation solves some problems addressed in the previous paragraph. Thermal energy can be stored for months or just for a couple of hours. There are various principles of heat storage with different properties, such as storage temperature level, energy density, efficiency, charge/discharge time, and cost^4,5^.

This paper focuses on a particular design of a latent heat thermal energy storage (LHTES) intended for demand side management (load shifting, peak shaving, valley filling). The application of phase change materials (PCMs) for heat accumulation is beneficial from the energy density perspective, allowing a smaller storage volume when compared with commonly used stratified water storage^6^. However, the downside of using PCM is that the PCM must be kept separated from the heat transfer fluid (HTF), which limits the charging/discharging times because PCMs are usually relatively poor heat conductors. Many heat transfer enhancement techniques have been proposed to tackle this problem^7,8^. A popular way is to add some materials like graphite powder, carbon fibers, graphene, or metal foam. However, these solutions appear prohibitively expensive or unreliable in long-term performance and are thus impractical for real-life applications.

Therefore, two types of more robust solutions are often used. The first one is to encapsulate the PCM into small containers of various shapes surrounded by the HTF^9–13^. The main advantage of this approach is technological simplicity, but at the cost of relatively low energy density and usually long charge/discharge times. Long charge/discharge times can be avoided by microencapsulation, where the interface area between the PCM and HTF is relatively large^9^. The second option is to use the inspiration from the heat exchanger design. Shell-and-tube LHTES^11^ usually offer high energy density, but they are lacking in heat transfer rate. Therefore, various types of LHTESs with fins protruding to the PCM have been designed^14–18^. Fins can increase the heat transfer rate significantly, but they are usually complicated to attach to the pipes reliably, which makes them expensive^19^. Many numerical studies using fins have been published, too^20^. However, they often focus too much on the optimal geometry without considering the manufacturing technology aspects of the design^21,22^.

A crucial step in designing an LHTES is the evaluation of its performance. The most common way of doing it is by charging or discharging the LHTES by constant flow rate and constant inlet temperature^12,16,17^. The outcome of this method is a high peak of maximum power (and outlet temperature) at the beginning of the process, followed by a gradual descent. This approach gives relevant data for numerical model validations, but it is very unlikely that the heat demand of a system will follow the gradually descending profile. Real charging/discharging is likely to be governed by advanced control algorithms like model-based predictive control (MPC)^23–25^.

Charging/discharging strategies that would emulate real-life conditions are rarely employed. For example, Nuytten et al.^9^ tested constant power charging/discharging of their LHTES designed for a CHP unit. However, they controlled the HTF inlet and outlet temperature difference while keeping a stable HTF flow rate, which might be impractical in real applications. Zauner et al.^26^ also reached almost constant charging power in their tests, but it was due to the power limit of their heating equipment. Xu et al.^27^ coupled their numerical model of an LHTES with a heating system model and compared various discharging strategies. Gradually increasing the flow rate through the LHTES was found to be the most effective. However, only numerical simulations supported the conclusion.

This article presents a demonstrator of a fin-and-tube LHTES. The performance of the prototype was evaluated employing a standard constant flow rate testing, and then a newly proposed constant mixing temperature method was tested on the demonstrator. Another part of the article deals with the mathematical modeling of the designed LHTES. The effective heat capacity approach was used for modeling the PCM. A lumped capacity model of the LHTES demonstrator was developed, and its heat transfer parameters were tuned using the constant flow experimental data. Finally, the analysis of the conducted experiments led to the execution of a set of simulations under different conditions, which showed the LHTES’s sensitivity to its power load.

One of the main contributions of this article to the ongoing research in the field of heat energy storage is the fin-and-tube LHTES demonstrator itself. It provides excellent stored energy density, which was found superior to previously published designs. At the same time, it has a decent power output rate, and it is easily scalable. The second contribution is the new constant mixing temperature method with a fixed total flow rate for storage performance evaluation. The experimental circuit for the method was designed, including control algorithms, and the experiments proved that the method worked as intended. The purpose of the method is to emulate real-life operation conditions. No similar approach was found in the literature. The third important part of the work is the lumped parameter simulation model. This kind of model was found to be highly time effective and precise when some heat transfer parameters were tuned using the experiments. The numerical model could be easily made a part of a simulation of a larger system. The fourth valuable result of the paper lies in the analysis of the experiments and the subsequent simulations. It showed that the LHTES would perform better in terms of its capacity utilization if it operated at lower power output than that experienced during the experiments. By application of this knowledge, a smaller, and thus more cost-effective, LHTES can be designed while the utility for the system remains the same.

## Experimental setup

### LHTES design

**Figure 1:**
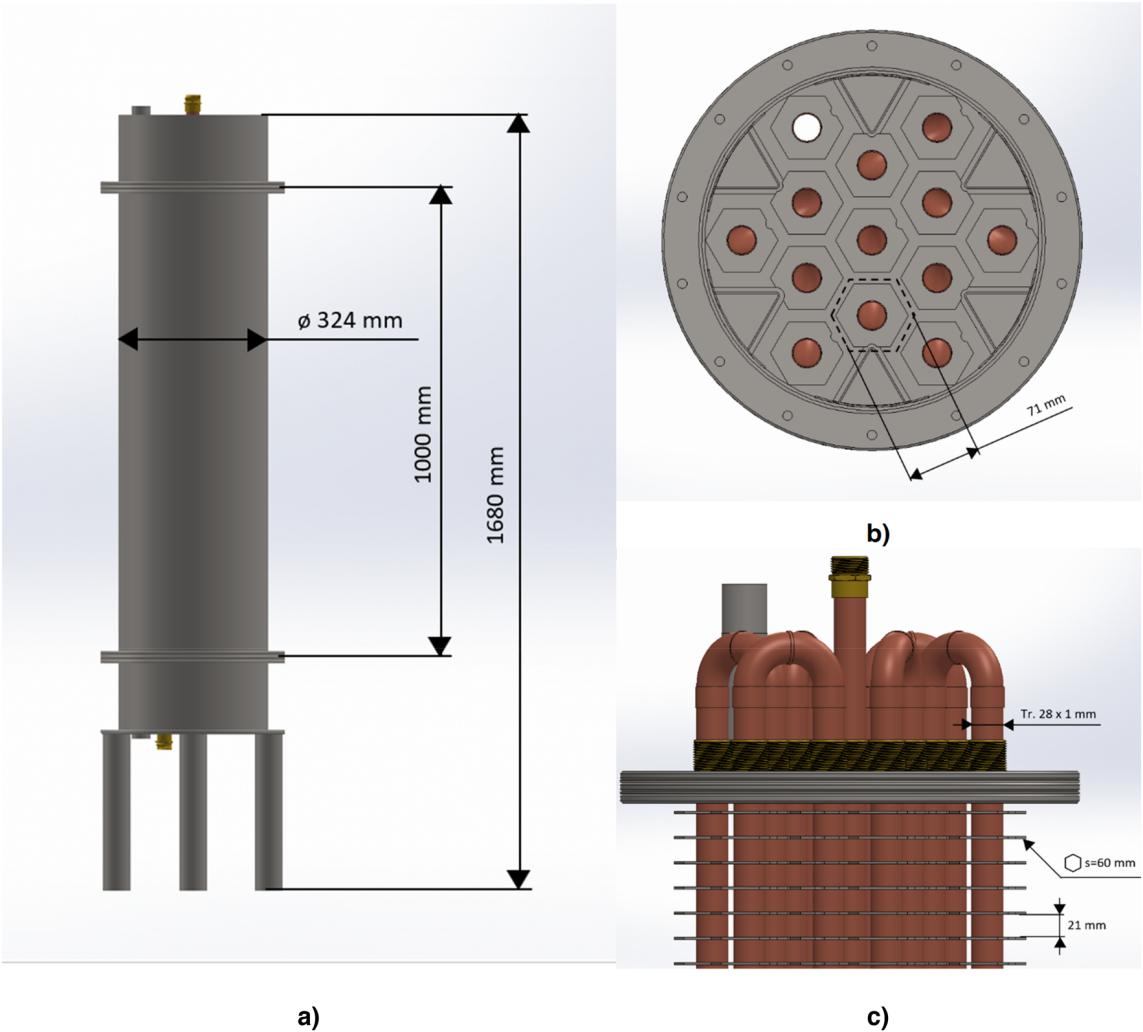
(**a**) Sideview of the storage and its general dimensions. (**b**) Top cut view of the storage; each of 13 tubes is surrounded by the equal volume of PCM; the outlet tube (11 o'clock position) is unobstructed, others are interconnected by “U” shaped fittings from the bottom. (**c**) The detail of the top part of the storage with relevant dimensions.

The design of the test device strictly follows the essential condition for the highly effective stored energy diffusion from the PCM areas to the walls of the tubing containing the flowing HTF. The main features of the design are displayed in Fig. 1. The internal space of the heat accumulator is uniformly structured for 13 sub-volumes of the hexagonal ground shape; the tube for heat transfer fluid is in the centroid of such sub-volume. Six “V” shaped spacers are equally installed round inside the cylindrical body of the main storage vessel to maintain the proper shape of those sub-volumes, which are close to the vessel wall.

The tubes for the heat transfer fluid are of an external diameter of 28 mm and a wall thickness of 1 mm. The material of the tubes is copper (EN 1057). The set of fins (spaced equally at a distance of 21 mm) of the hexagonal shape made from stainless metal sheets of 1,5 mm thickness is firmly connected to the cooper tubes to provide intensive heat conduction from accumulation areas to the tube walls. There are dents on the fins to prevent contact between the fins and the “V” shaped spacers. All fins are dented the same way for simple manufacturing. The dents have virtually no influence on heat transfer. The storage vessel contains 42,5 kg of PCM. The space for the heat storage is delimited by the cylindrical vessel body with the “V” shaped spacers from the sides and by the flat round lids from the bottom and the top.

A set of 13 tubes comes through the top and bottom lids via brass bushing parts; tubes are firmly connected to the bushing bodies which are part of the bottom lid. The tubes inside of the bushing parts installed on the top lid are axially moveable as the provision for tube thermal expandability effect elimination. On both ends, the system of tubes is mutually interconnected by the “U” shaped fittings into one flow line.

The internal space of the heat accumulator is filled with the PCM via the feed port located on the top lid; the PCM content is drained via the exhaust port located on the bottom lid. For the lab testing, the whole thermal storage vessel assembly is duly thermally insulated from the outside.

### LHTES comparison

Many latent heat storages have been designed. Therefore, a brief comparison with some previously proposed storages is presented in Table 1. It is not straightforward to compare various designs because they differ in size, PCM used, testing temperatures, flow rate, etc. Usually, the main motivation for considering LHTES is their high volumetric stored energy density. Therefore, it is the key parameter in the comparison.

**Table 1:** Comparison of the current design with previously published ones.

Authors	Design description	PCM (manufacturer)	T_min_/T_max_ (°C)	Energy density (kWh m^-3^)
This work	Fin-tube, PCM on fin-side	RT35HC (Rubitherm)	15/55	64.2
Jančík et al.^12^	Cylindrically encapsulated PCM	RT35HC (Rubitherm)	25/55	47.6
Xu et al.^27^	Cylindrically encapsulated PCM	C58 (Climator)	35/65	43.7
Zauner et al.^26^	Fin-tube, PCM on fin-side	HDPE (Ineos)	105/155	66.3
Khan et al.^17^	Fin-tube, PCM on fin-side	RT44HC (Rubitherm)	10/70	54.4
Kabbara et al.^15^	Fin-tube, PCM on fin-side	Lauric acid (Alfa Aesar)	20/70	63.5
Nuytten et al.^9^	Cylindrically encapsulated PCM	S58 (PCM Products)	43/73	55.1
Nuytten et al.^9^	Microencapsulated PCM	52 (Microtek Laboratories)	37/67	45.3

The current design achieved an energy density of $64.2{\text{ kWh}} \cdot {\text{m}}^{ - 3}$. That is comparable to the designs by Zauner et al.^16^ and Kabbara et al.^15^. However, both designs employed higher temperature differences, which is favorable. Therefore, the current design would perform better than these for the same temperature range in the field of energy density. Moreover, Kabbara et al. used unevenly located fins, leading to prohibitively long charging/discharging periods. In general, LHTESs with encapsulated PCM tend to have lower energy density. It is mostly due to a relatively larger portion of the HTF and capsules, which are poor heat-storing media.

### Experimental circuit and operation modes

**Figure 2:**
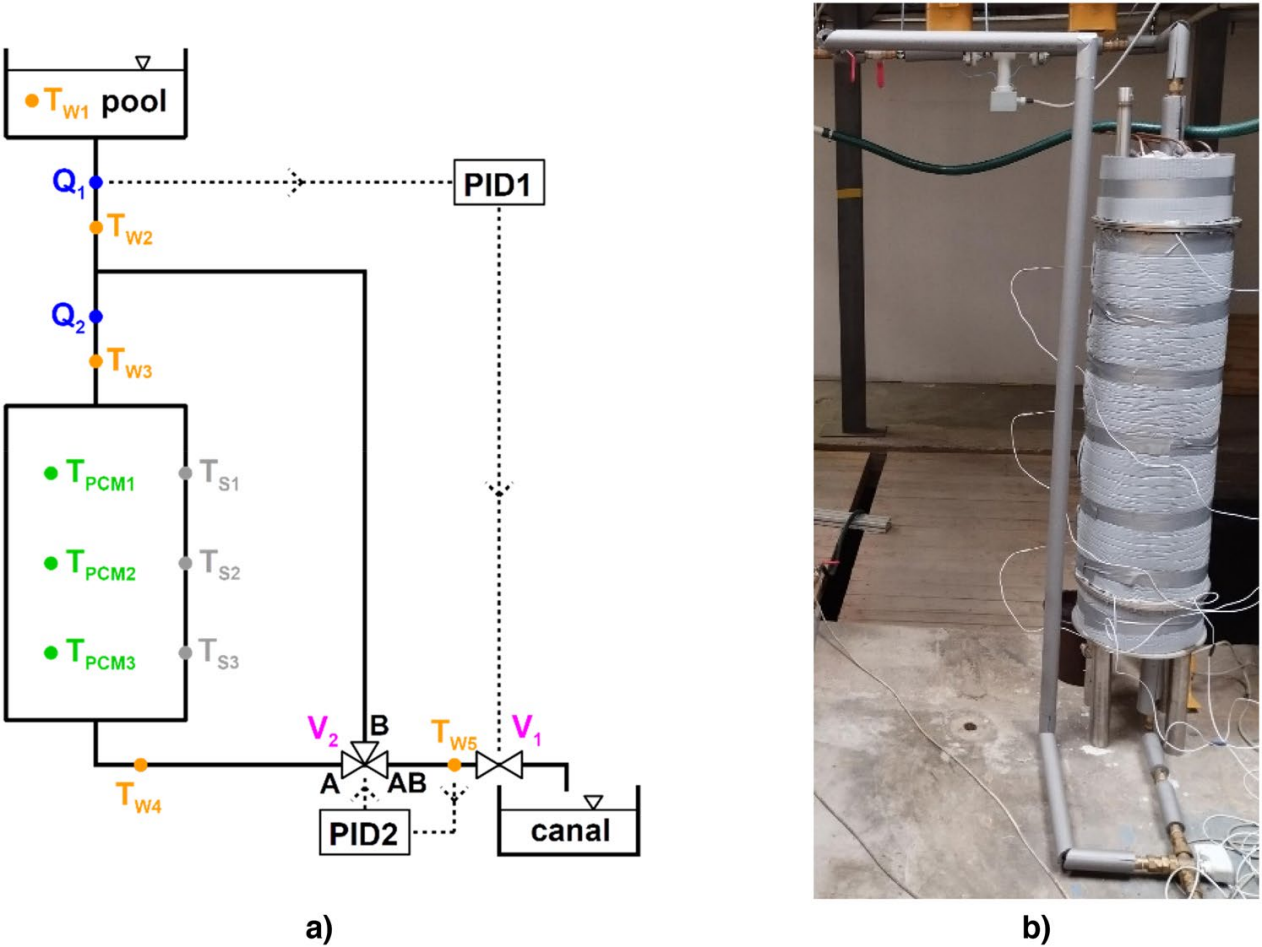
(**a**) The schematic of the experimental circuit for the LHTES with positions of sensors and control elements. (**b**) The actual LHTES connected into the system.

The LHTES was integrated into a circuit that allowed various modes of operation and data collection (Fig. 2). For the sake of clarity, only parts of the circuit and instruments relevant to the presented experiments are depicted. Before every experiment, the LHTES was heated with water warmed up by an electric heater in a closed loop (not shown in Fig. 2). The level of charge was checked by three temperature sensors placed directly in PCM (T_PCM1_, T_PCM2_, T_PCM3_) and three temperature sensors installed on the outer surface of the storage (T_S1_, T_S2_, T_S3_). Once they all reached the desired temperature (with $\pm 1\,^\circ {\text{C}}$ tolerance), the storage charging process terminated. There are two large water tanks: the upper one serves as the source of water at constant temperature (measured by T_W1_), and the lower one collects the water leaving the system.

Two modes of operation are presented in this work: constant flow rate through the storage and constant mixing temperature (with a constant flow rate through the whole system).

The constant flow rate experiments started with charged storage and mixing valve V2 set to fully closed (B-AB opened). Then valve V1 control algorithm was activated, and after about 60 s, when the flow rate measured by Q1 reached the required value, valve V2 was set to fully open (A-AB opened). The change in setting V2 takes about 60 s, and valve V1 compensates for any changes in system hydraulics, so the flow remains constant during the valve member position change and afterward.

The constant mixing temperature experiments started with charged storage and mixing valve V2 set to fully closed (B-AB opened). Then valve V1 control algorithm was activated, and after the flow rate measured by Q1 reached the required value, valve V2 was set to 20% open. After about 30 s, the control algorithm for valve V2 was activated and kept the mixed temperature ($T_{W5}$) at the desired value. The V2 control algorithm could not be activated when V2 was fully closed because it had resulted in large oscillations in the mixed temperature.

The valve control algorithms were two independent elements, both designed as PI controllers in LabVIEW. The idea of the V1 algorithm was simple: restrict the flow less when the valve when flow rate $Q_{1}$ is lower than desired and restrict it more when $Q_{1}$ is higher than desired. The idea of the V2 algorithm was similar: let more warm water flow from port A when temperature $T_{W5}$ is lower than desired and let more cold water flow from port B when $T_{W5}$ is higher than desired.

The gain and the integration time constants had to be set for both controllers. The installed valves integrated with the hydraulic system were nonlinear in their response, so the gain had to be the function of the current position of the valve member for a proper function of the whole system. The gain was a piecewise linear function found by measuring the characteristics of the valves prior to the herein-presented experiments. Cohen-Coon tuning method was used to find the appropriate parameters.

The heat power output of the storage was calculated using the following equation

1 $$P = \rho_{W} c_{pW} Q_{1} \left( {T_{W5} - T_{W2} } \right) ,$$

where $\rho_{W}$ and $c_{pW}$ are the density and the heat capacity of water, respectively (at temperature $T_{W2}$). Alternatively, it could be obtained using flow rate $Q_{2}$ with temperatures $T_{W3}$ and $T_{W4}$, but in the constant mixing temperature measurements, $Q_{2}$ is considerably smaller than $Q_{1}$ and thus less precisely measured.

### Instrumentation

The experimental storage was mounted with temperature probes for the measurement of water temperature in the inlet, the outlet, and the water pool. For monitoring the phase change, 3 temperature probes were placed inside the heat storage. This measurement was conducted using general-purpose RTD probes – 4-wire Pt 100. As an additional measurement, the temperature of the storage outer wall was measured at three different places using a 3-wire surface RTD probe Pt 100. Since the surface temperature measurement was there only for the confirmation of the fully charged state at the start of the experiments, and the values did not affect any calculations, simpler but fully appropriate sensors may be used for the purpose. All temperature probes were declared by the manufacturer to be of Class A accuracy per IEC60751, which means the tolerance $\pm 0.15 \,^\circ {\text{C}}$ at $0\, ^\circ {\text{C}}$ and $\pm 0.35 \,^\circ {\text{C}}$ at $100\, ^\circ {\text{C}}$.

The volumetric flow rate of water was measured by two electromagnetic flowmeters ELIS FLONET FN2014.1. These flowmeters were calibrated for the desired range of volumetric flow rates. The analog current signal was transmitted to the data acquisition (DAQ) unit.

The volumetric flow rate was controlled by one valve and one three-way valve. The two valves were driven by electromagnetic actuators SIEMENS SSB61, which were controlled by the voltage signal from the data acquisition and control unit.

As the data acquisition and control unit, the NI cDAQ 9188 with NI 9216 Temperature Input Module, NI 9203 Current Input Module, and NI 9263 Voltage Output Module were chosen. The measured data were preprocessed and stored using a PC which was connected via an Ethernet link to the main data acquisition and control unit. The software for the control and DAQ was created in LabVIEW. The temperature was recorded at a rate of $0.625 \,{\text{Hz}}$, and the flow rate was recorded at a frequency of $40 \,{\text{Hz}}$. The control loop for the constant heat power consisted of an advanced PID controller.

## Modeling

### PCM modeling

Melting or solidification can occur under a single temperature or within a temperature interval. The latter case relates to typical PCM, where enthalpy varies continuously within a mushy zone. A phase change is limited by solidus temperature in the lower limit and by liquid temperature in the upper limit. There coexist both solid and liquid phases in the mushy zone^28^. Most simple models of phase transition assume that the liquid phase mass fraction is a direct function of temperature in the mushy zone^29^. The liquid phase mass fraction is defined as

2 $$\zeta \left( T \right) = \frac{{m_{l} }}{{m_{l} + m_{s} }},$$

where $m_{l}$ is mass of a liquid phase and $m_{s}$ is mass of a solid phase. It is possible to write the enthalpy temperature relation in the form^29^

3 $$h\left( T \right) = \mathop \smallint \limits_{{T_{ref} }}^{T} \left( {\zeta \left( \tau \right)c_{l} + \left( {1 - \zeta \left( \tau \right)} \right)c_{s} } \right)d\tau + \zeta \left( T \right)\lambda ,$$

where $c_{l}$ and $c_{s}$ are specific heat capacities of liquid and solid phases, respectively, $\lambda$ is the latent heat of fusion. The reference temperature $T_{ref}$ for which the specific enthalpy is defined as zero is assumed to be much smaller than the temperature of phase transition. The enthalpy derivative according to temperature leads to the effective specific heat capacity of PCM

4 $$c_{eff} \left( T \right) = \zeta \left( T \right)c_{l} + \left( {1 - \zeta \left( T \right)} \right)c_{s} + \frac{\partial \zeta }{{\partial T}}\lambda .$$

The effective specific heat capacity model needs liquid phase mass fraction as a function of temperature. This function can be obtained by fitting experimental partial enthalpy data provided by the PCM manufacturer. The crucial step is the selection of this monotonously increasing function that realizes a smooth transition from 0 to 1. An unsymmetric probability density function fits the requirements**.** The Gumbel minimum cumulative distribution function is used in this model

5 $$\zeta_{i} = 1 - e^{{ - e^{{\frac{{T - \mu_{i} }}{{\beta_{i} }}}} }} ,$$

where $\mu_{i}$ is the localization parameter and $\beta_{i}$ is the scale parameter of the distribution. The Gumbel minimum probability density function is^29^

6 $$\frac{{d\zeta_{i} }}{dT} = \frac{1}{\beta }e^{{\frac{{T - \mu_{i} }}{{\beta_{i} }}}} e^{{ - e^{{\frac{{T - \mu_{i} }}{{\beta_{i} }}}} }} .$$

It is convenient to model the distribution of real PCM as a superposition of cumulative distribution functions

7 $$\zeta \left( T \right) = \mathop \sum \limits_{i = 1}^{n} w_{i} \zeta_{i} .$$

and probability distribution functions^29^

8 $$\frac{d\zeta }{{dT}} = \mathop \sum \limits_{i = 1}^{n} w_{i} \frac{{d\zeta_{i} }}{dT},$$

where each $\frac{{d\zeta_{i} }}{dT}$ has its parameters $\mu_{i}$ and $\beta_{i}$. The weighting factors are positive $w_{i} > 0$ with $\sum\nolimits_{i = 1}^{n} {w_{i} = 1}$. A nonlinear interpolation technique is used to find the parameters. The superposition of two Gumbel probability density (cumulative distribution) functions is used to approximate the properties of PCM in this work.

**Figure 3:**
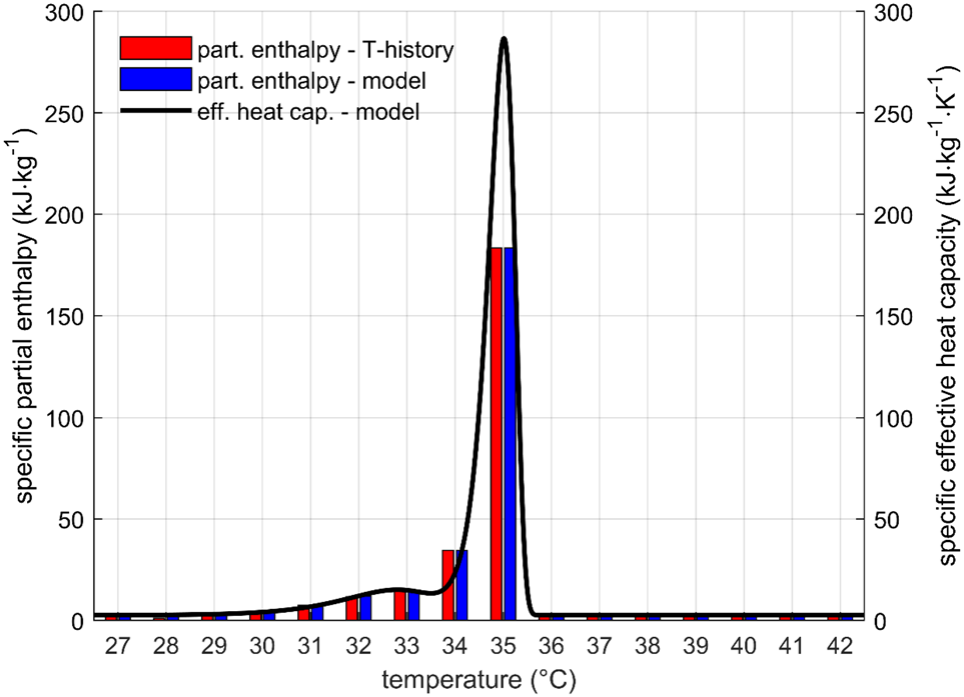
RT35HC—Comparison of partial enthalpy measured by T-history method, effective heat capacity model and respective partial enthalpy. Parameters of the model were successfully fitted to the measured data.

RT35HC from Rubitherm^30^ was used as the PCM in the LHTES. Specific partial enthalpy was obtained by the T-history method and its details have been previously published^12^. Figure 3 shows the partial enthalpy from the experiment, the fitted effective heat capacity and the partial enthalpy corresponding to the fit. The reasons for using a PCM with relatively low phase change temperature were lab environment safety and lower energy consumption for melting due to the smaller difference between the maximal temperature and the ambient. The authors are confident that the results with this PCM can be transposed to higher temperatures as long as the relative differences between the relevant temperatures are preserved.

### Heat storage modeling

A numerical storage model was developed. The priorities of the modeling were computational effectivity, simple scalability for solving larger storages, and the ability to be part of more complex models. To get all the listed properties, a lumped parameter technique was chosen. The authors presented a similar approach in^12^. The advantages of this type of modeling are listed above, but there are also disadvantages. For example, heat transfer between PCM and the heat transfer surface (pipe surface and ribs) is complicated, and some of its parameters must be found by fitting the model with experimental data.

The necessary parameters of the model were tuned using the constant flow rate experiments (In "Constant flow rate" section). The model was then validated by comparison of the simulation with the data from the constant mixing temperature with fixed total flow rate experiments (In "Constant mixing temperature with fixed total flow rate" section). See the respective sections to assess the level of accuracy of the model.

**Figure 4:**
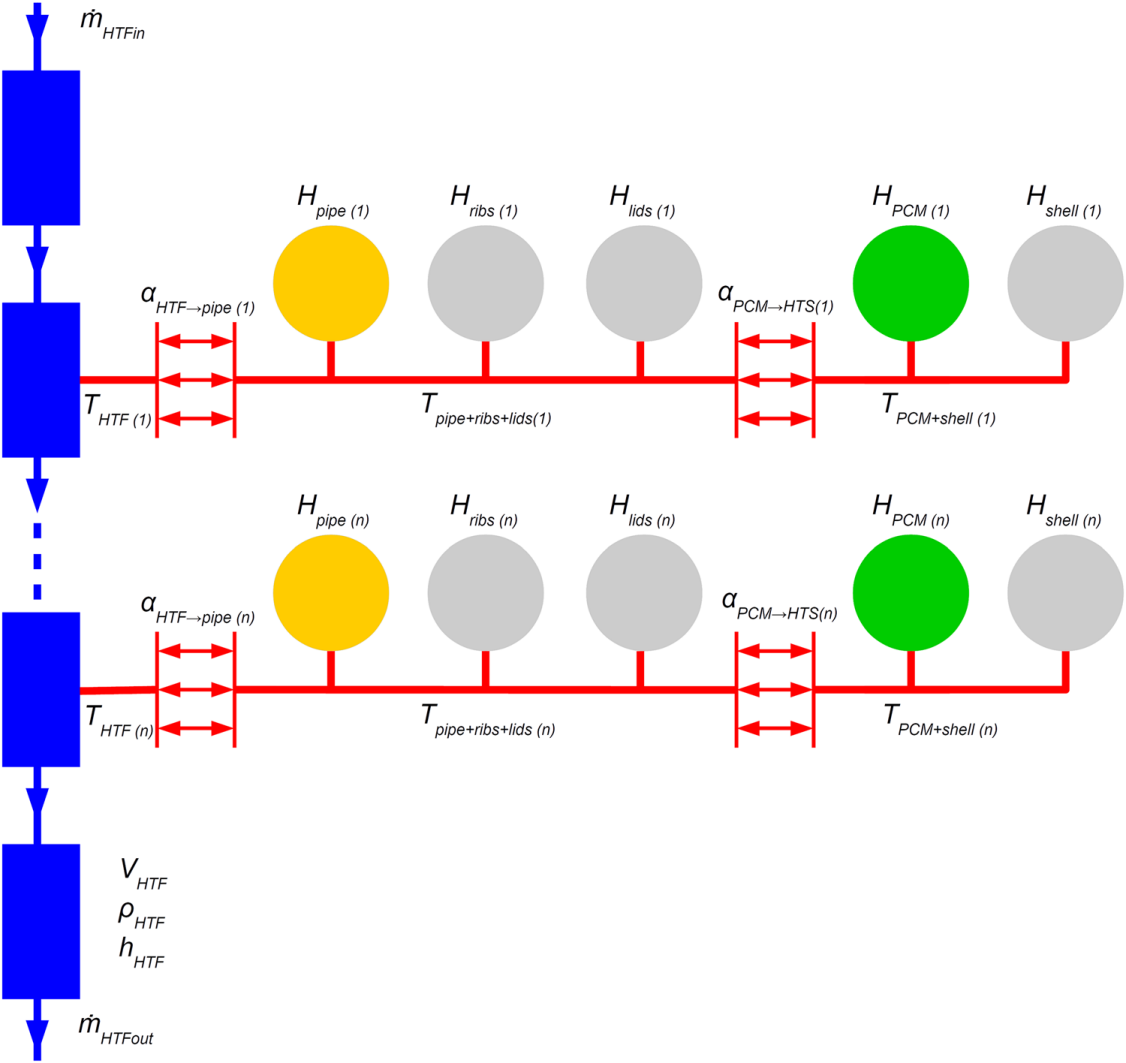
Numerical model schematic. HTF volumes—blue; heat capacitors—orange, gray, and green (material dependent); heat transfer components—red.

The schematic of the model is shown in Fig. 4. The model consists of three types of elements: heat transfer fluid volumes, heat capacitors, and heat transfer components. These elements are interconnected so that the heat fluxes and changes in the state of the materials are represented accurately while keeping the model topology simple.

The HTF inside the pipe is represented by a chain of control volumes. The HTF has constant density, so the mass balance is simply

9 $$\dot{m}_{HTFin} = \dot{m}_{HTFout} .$$

Specific enthalpy in the volume is balanced using the following equation

10 $$\frac{{{\text{d}}\left( {h_{HTF} } \right)}}{{{\text{d}}t}} = \frac{1}{{V_{HTF} \rho_{HTF} }}(\dot{m}_{HTFin} h_{HTFin} - \dot{m}_{HTFout} h_{HTF} + \dot{Q}_{in} ) ,$$

where the first two terms on the right-hand side of the equation represent convective transport of enthalpy, and the last one is heat flux through the wall of the volume. Enthalpy is a function of temperature.

There is no heat transfer in the first volume and the last one because they represent the short portions of pipe that are between the inlet and the inlet temperature sensor and between the outlet and the outlet temperature sensor, respectively. These short parts proved functional, especially at the beginning of the simulations, where they improved the dynamic behavior of the storage model.

Between the first and the last volumes, there is a series of *n* interconnected volumes that take part in heat transfer. Each of these represents an equal portion of the pipe inside the storage. Heat transfer between the HTF and the inner wall of the pipe is described by Newton’s cooling law

11 $$\dot{Q}_{HTF \to pipe} = {\upalpha }_{HTF \to pipe} \pi d_{pipe in} \frac{{{\text{L}}_{pipe} }}{n}\left( {T_{HTF} - T_{pipe + ribs + lids} } \right),$$

where the heat transfer coefficient is calculated from the correlation

12 $${\upalpha }_{HTF \to pipe} = \frac{{k_{HTF} \cdot {\text{Nu}}}}{{d_{pipe in} }} ,$$

where $k_{HTF}$ is the heat conductivity of the HTF and ${\text{Nu}}$ is the Nusselt number. For lower ${\text{Re}}$ (laminar flow, ${\text{Re}} \le 2300$), U-bends affect heat transfer. The flow develops thermally after each bend^31^, so an appropriate correlation should be used. For thermally developing flows, the determining parameter is nondimensional pipe length, defined as

13 $$x^{*} = \frac{{L_{seg} }}{{d_{pipe in} \cdot {\text{Re}} \cdot {\text{Pr}}}} ,$$

where $L_{seg}$ is the length of the pipe between two bends (1 m in the modelled case). The used correlation for ${\text{Nu}}$ is

14 $${\text{Nu}}_{lam} = \left( {3.66^{3} + \frac{{1.19x^{* - 0.8} }}{{1 + 0.117x^{* - 0.467} }}} \right) ,$$

which is valid for $0.0001 \le x^{*} \le 10$ [waermeatlas]. For higher ${\text{Re}}$, thermal development of the flow is not significant, so standard correlations for transition and fully turbulent flow are used^32^. The whole range of ${\text{Re}}$ is covered by the following relations

15 $$\begin{array}{*{20}l} {{\text{if }}\,\,{\text{Re}} \le 2300} \hfill & {\left\{ {{\text{Nu}} = {\text{Nu}}_{lam} } \right.} \hfill \\ {{\text{if}}\,\,{ }2300 \le {\text{Re}} \le 10^{4} } \hfill & {\left\{ {\begin{array}{*{20}l} {{\text{Nu}} = \left( {1 - \gamma } \right) \cdot {\text{Nu}}_{lam} + \gamma \frac{{\frac{\zeta }{8} \cdot 10^{4} \cdot {\text{Pr}}}}{{1 + 12.7\sqrt {\frac{\zeta }{8}} \cdot \left( {{\text{Pr}}^{\frac{2}{3}} - 1} \right)}}} \hfill \\ {\gamma = \frac{{{\text{Re}} - 2300}}{{10^{4} - 2300}}} \hfill \\ {\zeta = \left[ {1.8\log \left( {10^{4} } \right) - 1.5} \right]^{ - 2} } \hfill \\ \end{array} } \right.} \hfill \\ {{\text{if}}\,\,{ }10^{4} \le {\text{Re}}} \hfill & {\left\{ {\begin{array}{*{20}l} {{\text{Nu}} = \frac{{\frac{\zeta }{8} \cdot {\text{Re}} \cdot {\text{Pr}}}}{{1 + 12.7\sqrt {\frac{\zeta }{8}} \cdot \left( {{\text{Pr}}^{\frac{2}{3}} - 1} \right)}}} \hfill \\ {\zeta = \left[ {1.8\log {\text{Re}} - 1.5} \right]^{ - 2} } \hfill \\ \end{array} } \right.} \hfill \\ \end{array}$$

The pipe itself, the ribs, and the lids through which the pipes go (or their fractions, depending on the chosen number of volumes) are represented by heat capacitors. Their enthalpy rate of change is given by

16 $$\frac{{{\text{d}}H}}{{{\text{d}}t}} = C\frac{{{\text{dT}}}}{{{\text{d}}t}} = \dot{Q}_{in} ,$$

where $C$ is heat capacity and $\dot{Q}_{in}$ is heat flux into the capacitor. These components are assumed to have identical temperatures because their interconnections are relatively well conductive. The heat capacity of steel and copper are considered constants.

**Figure 5:**
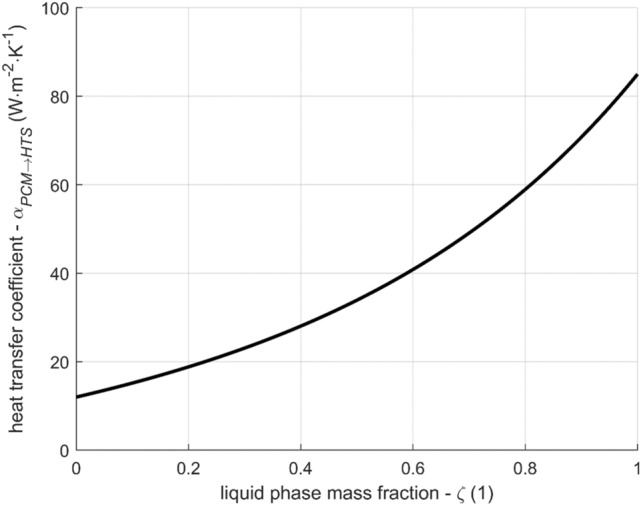
Heat transfer coefficient between PCM and heat transfer surface as a function of liquid phase mass fraction according to Eq. (17).

The heat transfer coefficient between the PCM and the heat transfer surfaces (pipe outer surface and ribs) is modeled as a function of liquid phase mass fraction. It respects the fact that the intensity of heat transfer depends on the state of the PCM. The authors employed a similar approach successfully before using a linear function^12^. A linear function yielded an acceptable outcome, but the results presented in this article use a function with one more parameter describing its shape. The chosen function is

17 $$\alpha_{PCM \to HTS} = \alpha_{solid} + \left( {\alpha_{liquid} - \alpha_{solid} } \right) \cdot \left\{ {{\text{exp}}\left[ {k\left( {\zeta - 1} \right)} \right] + {\text{exp}}\left( { - k} \right) \cdot \left( {\zeta - 1} \right)} \right\} ,$$

where $\alpha_{solid}$ and $\alpha_{liquid}$ are heat transfer coefficient for $\zeta = 0$ and $\zeta = 1$, respectively and $k$ is the parameter influencing the shape of the function. The three parameters were found by fitting the results of the model with the measured data for constant flow rate, yielding the result $\alpha_{solid}$ = 12 ${\text{W}} \cdot {\text{m}}^{2} \cdot {\text{K}}^{ - 1}$, $\alpha_{liquid} = 85{\text{ W}} \cdot {\text{m}}^{2} \cdot {\text{K}}^{ - 1}$, and $k = 2$. The resulting curve is shown in Fig. 5.

The reason for the form of the function in Eq. (17) can be a gradually increasing thickness of the solid PCM layer deposited on the heat transfer surfaces as the PCM solidifies. The thermal conductance of the layer is indirectly proportional to its thickness, which is proportional to $\left( {\zeta - 1} \right)$. There is also a heat resistance independent of the PCM state, so the overall conductivity is finite even for $\zeta = 1$. This reasoning leads to a function $\alpha_{PCM \to HTS}$ that is convex and growing with the liquid mass fraction $\zeta$.

PCM is modeled according to Equation ( 16) with heat capacity $C$ as a product of PCM mass (or its fraction, depending on the chosen number of volumes) and its specific effective heat capacity according to Equation ( 4). The outer shell of the storage has the same temperature as the PCM since it has direct contact with the PCM.

An important numerical parameter is the previously mentioned number of volumes of HTF and respective sections of other components. A simple convergence study was performed, and there was virtually no difference in results for five and more volumes (for a pipe length of about 13 m). The further presented results are for 15 volumes because the extra computational cost was found negligible.

### State of charge

One of the methods used in^12^ was used for the evaluation of the state of charge (SoC) of the storage. For discharging it is

18 $$SoC\left( {\text{t}} \right) = 1 - \frac{{\mathop \smallint \nolimits_{0}^{t} P\left( \tau \right)d\tau }}{{{\Delta }H_{max} }} ,$$

where $P$ is the storage power output defined by Equation ( 1) and ${\Delta }H_{max}$ is the difference in enthalpy between the storage at the maximum and the minimum temperature. For the investigated storage, it is

19 $${\Delta }H_{max} = \mathop \smallint \limits_{{T_{min} }}^{{T_{max} }} \left( {c_{eff} \left( \theta \right) \cdot m_{PCM} + c_{HTF} \cdot m_{HTF} + C_{pipe} + C_{ribs + lids + shell} } \right){\text{d}}\theta ,$$

where masses of the PCM, HTF, and components are known from the design of the storage and specific heat capacities of copper and stainless steel are taken as constants. For every case in this study, $T_{max}$ and $T_{min}$ were taken as the initial temperature of the storage at the beginning of the discharging period $T_{ini}$ and the inlet temperature of the HTF $T_{in}$, respectively.

## Results and discussion

### Constant flow rate

Constant flow rate is a standard heat storage test, which was performed with many designs many times, for example, in^10,16,26^. In this work, two flow rates were measured (0.9 m^3^/h and 0.45 m^3^/h) with the initial storage temperature (measured by sensors T_PCM1_, T_PCM2_, T_PCM3_ T_S1_, T_S2_, and T_S3_) close to 55 °C and the inlet water temperature of about 15 °C.

**Figure 6:**
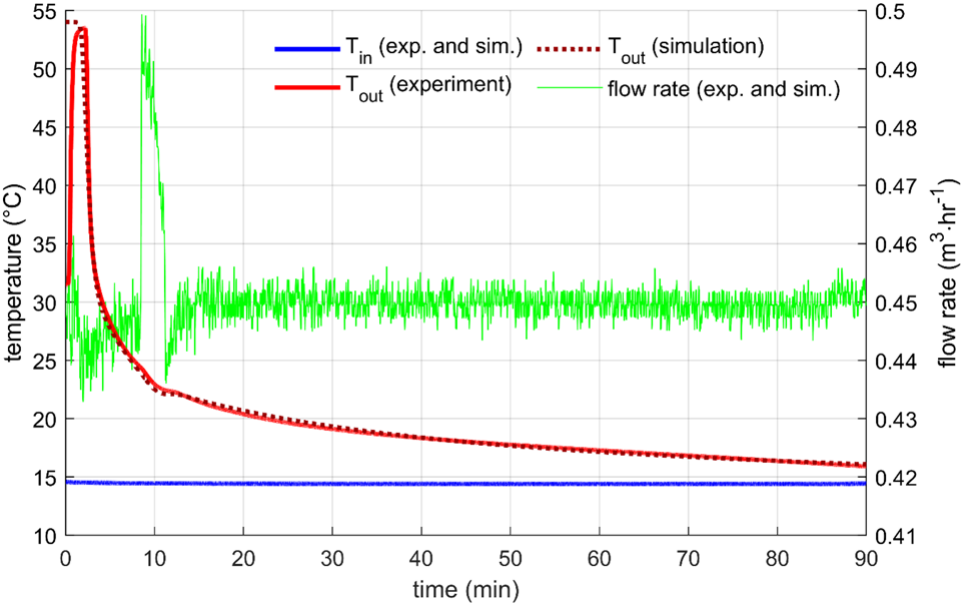
Performance parameters for constant flow rate 0.45m3/h—results of the experiment and the simulation.

**Figure 7:**
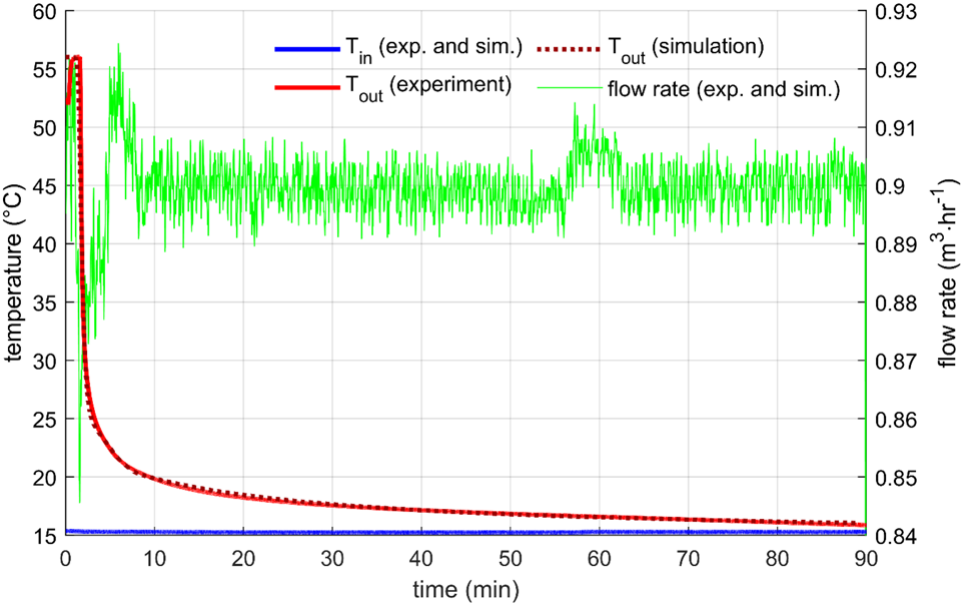
Performance parameters for constant flow rate 0.9 m3/h—results of the experiment and the simulation.

In both cases, the outlet temperature profile was very similar (Figs. 6 and 7). The outlet temperature was equal to the initial storage temperature for a short period as the warmer water from the storage was substituted by inflowing colder water. After that, the outlet temperature was getting colder relatively quickly. Naturally, the discharge process was faster for the higher flow rate. The flow rate through the storage was controlled and was kept within a 1% error from the desired value for most of the time. Few more significant flow fluctuations occurred during the experiments, but they did not affect the overall outcome. The cause of the fluctuations is unknown; possible water contamination with impurities might cause a sudden change in the hydraulic characteristic of the system.

The parameters for the numerical description of heat transfer between the PCM and the heat transfer surfaces were found by fitting with the experimental data, as mentioned in the previous section. For both flow rates, the level of agreement was excellent, which indicates an appropriate choice of the structure of the heat transfer function for the studied configuration [Eq. (17)].

### Constant mixing temperature with fixed total flow rate

Constant mixing temperature experiments were designed to test the storage performance in a scenario that is closer to real-life operation conditions than the constant flow rate. Four settings were measured: Outlet temperatures 20 °C and 25 °C with total flow rates of 0.45 m^3^/h and 0.9 m^3^/h. Only three of them are presented here because for the highest power settings (25 °C and 0.9 m^3^/h), the used control algorithms and hardware could not reach the desired outlet temperature for a technically relevant period. This experiment showed the limits of the used hardware and algorithms. For the tested setup, the maximal reasonable power setting was found to be around 8 kW, which is close to case 2 experiment.

**Figure 8:**
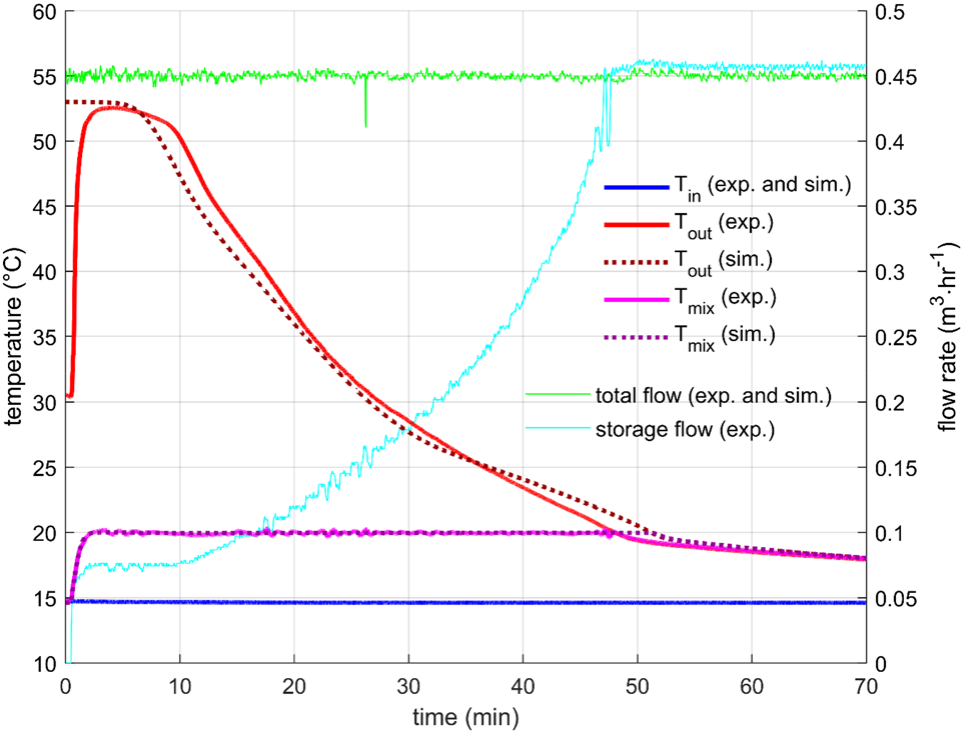
Performance parameters for constant total flow rate 0.45m3/h and desired mixing temperature 20 °C—results of the experiment and the simulation.

**Figure 9:**
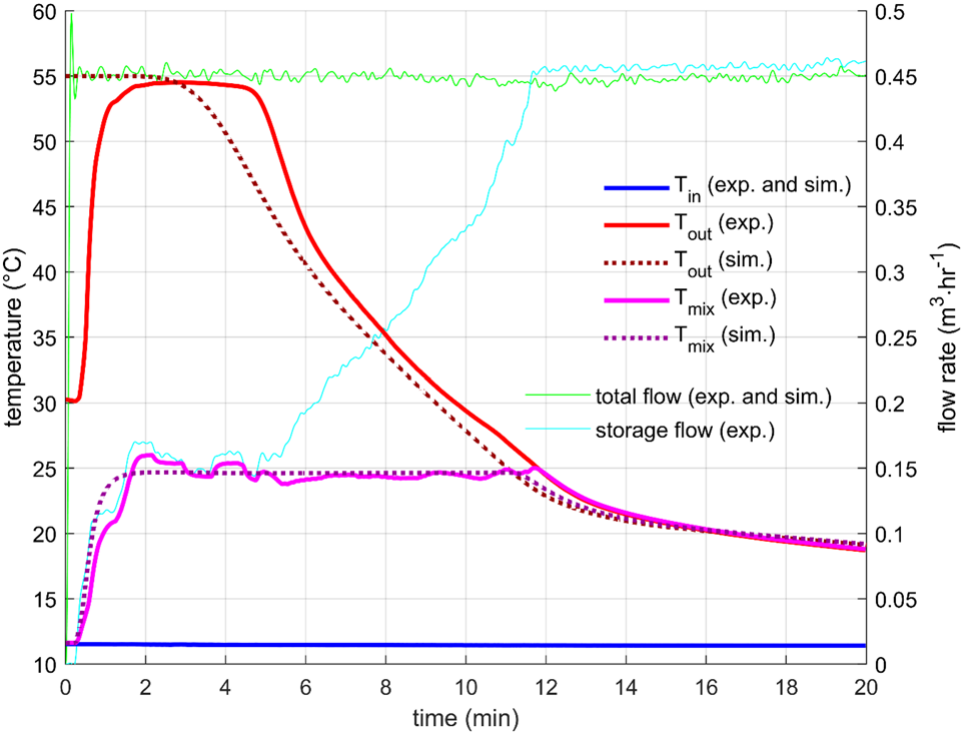
Performance parameters for constant total flow rate 0.45m3/h and desired mixing temperature 25 °C—results of the experiment and the simulation.

**Figure 10:**
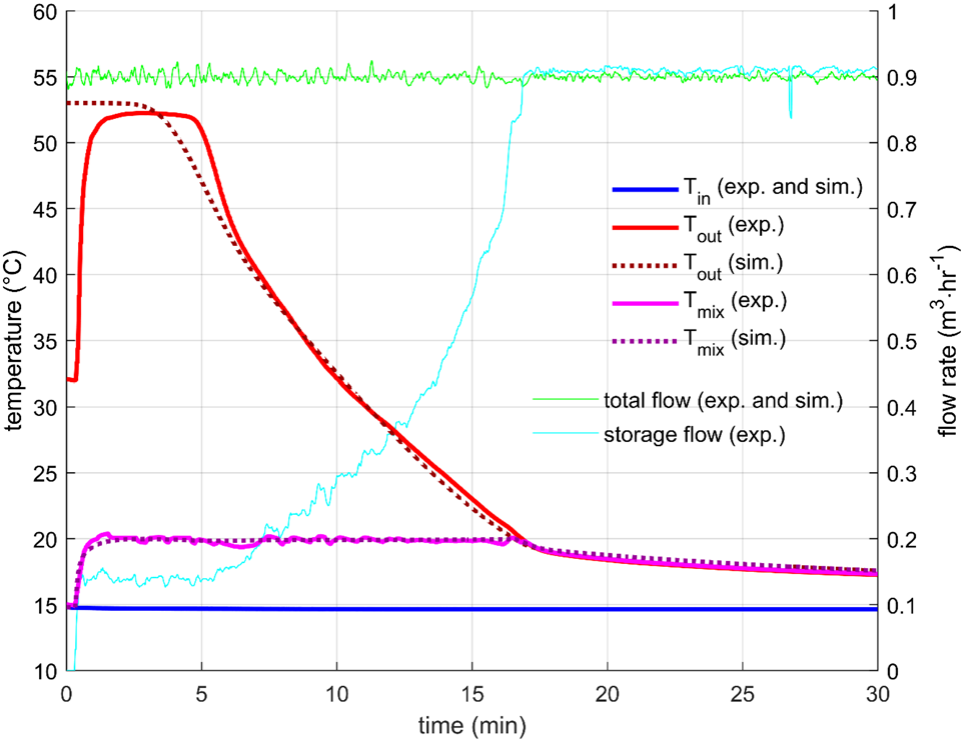
Performance parameters for constant total flow rate 0.9 m3/h and desired mixing temperature 20 °C—results of the experiment and the simulation.

The results are presented in Figs. 8, 9, and 10. The starting transition period took about two minutes for the lower flow rate and about one minute for the higher flow rate. After that time, the controlling mixing valve was able to keep the outlet temperature very close to the desired value. The faster the rate of change of the outflow temperature, the greater the control error. However, it was well below 1 °C for all presented cases, which is more than satisfactory for the intended purpose.

The total flow through the system was kept constant during the experiments with no extensive fluctuations. The flow rates through the storage are presented in Figs. 8, 9, and 10 as well. A gradual flow rate growth was occurring as the outlet temperature was decreasing. A similar flow rate profile was proposed by Xu et al.^27^, and their simulations indicated that it is close to the ideal flow rate profile for discharging an LHTES. The experimental investigation presented here thus confirms their findings from numerical simulations.

**Figure 11:**
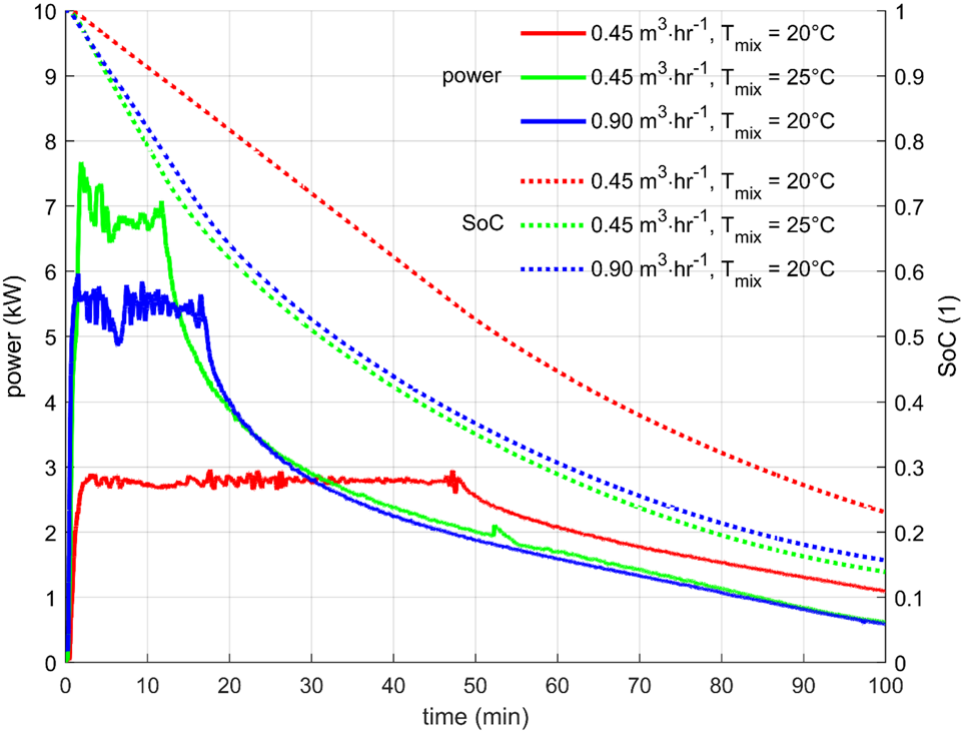
The comparison of the output power and the state of charge for the three cases of constant mixing temperature.

The comparison between the cases is presented in Fig. 11, and the summary is in Table 2. It is seen that the higher the desired power, the shorter the time spent at that power. The results demonstrate the importance of the power output of the storage to its ability to use the heat at the desired temperature level. Compare, for example, case 1 and case 3, which worked with virtually identical temperatures. Regarding the power output, case 3 gave about twice the power compared to case 1. However, discharging in case 1 lasted almost three times longer. It led to an almost 50% increase in heat released at the desired temperature. The state of charge (SoC) also confirmed this finding, as 45% of the stored heat was used in case 1 and only 30% in case 3.

**Table 2:** The summary of the parameters for constant mixing temperature cases.

Case	T_ini_ (°C)	T_in_ (°C)	T_mix_ (°C)	Flow (m^3^ hr^-1^)	Power (kW)	Time (min)	Heat (kWh)	SoC (1)
1	53	15	20	0.45	2.8	46	2.15	0.55
2	55	12	25	0.45	6.9	11	1.27	0.75
3	53	15	20	0.90	5.4	16	1.44	0.70

Data from the experiments. The values are rounded.

Of course, the heat released after the mixed temperature drops below the desired temperature is not lost, but it is of less utility because of its lower potential. It can be used only for pre-heating, and some other measures must be taken, like starting a conventional heating or another charged heat storage.

The dominant reason for the fact that the faster the storage charges/discharges, the less portion of its capacity can be effectively used is the limited heat transfer rate within the storage, particularly in the PCM. Unfortunately, changes in the design leading to heat transfer enhancement (smaller capsules, increased number of fins, etc.) are usually costly and decrease the heat storage energy density^9,11^.

The range of parameters investigated by the experiment seemed inappropriate for the designed LHTES. The SoC at the end of the discharging period was too high, and most of the stored heat was not extracted. Therefore, simulations of discharging at lower power settings were performed. The goal was to find if lowering the power output would lead to an appropriate discharge time and if the capacity of the storage is utilized sufficiently, i.e., if the SoC at the end of the discharge is adequately low.

The parameters and the results of the simulations are summarized in Table 3. Firstly, there was an effect of desired output temperature (T_mix_). Even though the power was the same in cases 4 and 6 and 5 and 7, respectively, higher T_mix_ led to lower heat utilization. The extracted heat was 23% lower for the higher power (2 kW), and it was 15% lower for the lower power (1 kW) when compared between T_mix_ = 20 °C and T_mix_ = 25 °C. Secondly, there was the desired output power effect. When comparing cases 4 and 5, there was a 29% increase in heat utilization, and for cases 6 and 7, there was an increase of 43%. Not surprisingly, the best heat utilization was found in case 5 with the least demanding conditions. However, the overall discharging time, in this case, was almost four hours, and it might be too much for some applications.

**Table 3:** The summary of the parameters for constant mixing temperature cases.

Case	T_ini_ (°C)	T_in_ (°C)	T_mix_ (°C)	Flow (m^3^ hr^-1^)	Power (kW)	Time (min)	Heat (kWh)	SoC (1)
4	55	15	20	0.35	2.0	90	3.00	0.37
5	55	15	20	0.18	1.0	233	3.88	0.21
6	55	15	25	0.18	2.0	69	2.30	0.53
7	55	15	25	0.09	1.0	198	3.30	0.33

Data from the simulations. The values are rounded.

Finding the optimal design of an LHTES for an intended application does not involve only finding a suitable capacity and phase change temperature of a PCM. The design has to respect the charging times and powers usually achieved in the system. Doing this can achieve significant savings in the required space, construction materials (including PCM), production hours, and, thus, costs. Making an LHTES that can exploit the maximum of its heat storage capacity at usable temperature levels and not making it unduly complicated and thus expensive is anything but a trivial task.

## Conclusion

The fin-and-tube LHTES presented in this article has an excellent value of energy density and a relatively simple scalable design. The comparison with other designs showed that, in general, fin-and-tube storages are superior to storages with capsules in terms of energy density, and the current design is among the best considering this parameter. Between temperature levels 15 °C and 55 °C it can store $64.2 \,{\text{kWh}}\,{\text{m}}^{ - 3}$.

A new constant mixing temperature test that is much closer to an actual LHTES operation mode was proposed and tested with the special laboratory circuit. The used algorithms for controlling temperature and flow rate proved to be sufficiently robust for the task. It was found the constant mixing temperature test is a useful tool for the evaluation of thermal storages at the stage of initial design in computer simulations and on laboratory prototypes for verification of their qualities.

The computational model based on the effective heat capacity PCM modeling and lumped heat capacity approach provided results that agreed with the data from the experiment very well. The computational simplicity of the model allows it to be used as part of more complex models of whole distribution systems. It is also easily expanded into greater scales, and more storages can be simulated together, possibly with different PCM phase change temperature levels.

The analysis of the constant mixing temperature tests revealed that the experimentally tested settings were not ideal for the LHTES, being too demanding on the power output and thus not exploiting the capacity of the storage effectively. Additional simulations illustrated how changes in performance parameters of the LHTES operation can improve the utilization of the stored heat. The amount of heat extracted from the storage at the desired temperature parameters was only between 25 and 45% for the experimental cases (cases 1–3). The simulations confirmed this finding. Additional simulations (cases 4–7) showed that between 47 and 79% of stored heat could be recuperated from the storage if the heat power demand was lower. While the output temperatures remained the same, obtained heat was almost double.

## Data Availability

The datasets used and analyzed during the current study available from the corresponding author on reasonable request.
